# Regulation of NF-*κ*B-Induced Inflammatory Signaling by Lipid Peroxidation-Derived Aldehydes

**DOI:** 10.1155/2013/690545

**Published:** 2013-04-17

**Authors:** Umesh C. S. Yadav, Kota V. Ramana

**Affiliations:** Department of Biochemistry and Molecular Biology, University of Texas Medical Branch, Galveston, TX 77555, USA

## Abstract

Oxidative stress plays a critical role in the pathophysiology of a wide range of diseases including cancer. This view has broadened significantly with the recent discoveries that reactive oxygen species initiated lipid peroxidation leads to the formation of potentially toxic lipid aldehyde species such as 4-hydroxy-trans-2-nonenal (HNE), acrolein, and malondialdehyde which activate various signaling intermediates that regulate cellular activity and dysfunction via a process called redox signaling. The lipid aldehyde species formed during synchronized enzymatic pathways result in the posttranslational modification of proteins and DNA leading to cytotoxicity and genotoxicty. Among the lipid aldehyde species, HNE has been widely accepted as a most toxic and abundant lipid aldehyde generated during lipid peroxidation. HNE and its glutathione conjugates have been shown to regulate redox-sensitive transcription factors such as NF-*κ*B and AP-1 via signaling through various protein kinase cascades. Activation of redox-sensitive transcription factors and their nuclear localization leads to transcriptional induction of several genes responsible for cell survival, differentiation, and death. In this review, we describe the mechanisms by which the lipid aldehydes transduce activation of NF-*κ*B signaling pathways that may help to develop therapeutic strategies for the prevention of a number of inflammatory diseases.

## 1. Introduction

Lipid peroxidation-derived aldehydes (LDAs) have been implicated in a number of oxidative stress-induced inflammatory pathologies such as diabetes, metabolic syndrome, vascular and neural degeneration, liver and kidney toxicity, cancer, retinopathy of prematurity, aging, and ischemia [1–12]. LDAs such as malondialdehyde (MDA), 4-hydroxy-2-nonenal (HNE), and acrolein are generated upon degradation of lipid peroxides subsequent to free radical-induced peroxidation of membrane lipids, particularly the polyunsaturated fatty acids, in the biological membranes [13, 14]. Arachidonic acid present in the biological membranes is predominantly susceptible to free radical attacks due to the presence of unsaturated bonds and is the primary source of LDAs. LDAs are relatively more stable as compared to free radicals such as oxygen and hydroxyl free radicals and act as highly reactive electrophilic molecules [13]. Quantitatively, while HNE and MDA are the most abundant aldehydes formed subsequent to lipid peroxidation, acrolein is the most reactive one. However, LDAs in general, have a tendency to react readily with the nucleophiles including thiols and amines containing cellular macromolecules such as proteins, and nucleic acids leading to cellular damage and accumulation of chemically altered macromolecules [15, 16]. In various disease states conjugates of LDAs with proteins and nucleic acids have been identified; for example, HNE-protein adducts were detected in mitotic, necrotic, and apoptotic cells in brain tumor tissues [17]. LDAs can act as toxic secondary messengers to propagate redox signals leading to cellular and tissue injury [18–20].

HNE generated from the peroxidation of arachidonic acid is highly toxic and the most abundant LDA in the living tissue reaching a reported concentration of up to 10 nanomol/g tissue [13]. Its in-situ concentration plays a key role in the cell growth, death, and differentiation. HNE has been shown to exert marked biological effects by affecting and altering cellular signaling events, modifying and damaging protein and DNA, eventually leading to cytotoxicity and pathogenesis [18, 19]. Similar effects have been reported for acrolein as well [21–23]. In this review, we have discussed the mechanism of production of LDAs and their roles in regulating the inflammatory signals that activate redox-sensitive transcription factors such as NF-*κ*B. 

## 2. Oxidative Stress and Lipid Peroxidation

In aerobes, varieties of highly reactive chemical entities are formed as the by-product of the oxygen utilization which is collectively termed as reactive oxygen species (ROS) [24, 25]. The ROS include superoxide anion (O_2_^−^), hydroxy radical (OH^·^), nitric oxide radical (NO^·^), and their by-products (e.g., hydrogen peroxide, H_2_O_2_). A constant flux of ROS caused by acute or chronic inflammatory diseases or environmental stresses leads to a state of moderately increased levels of intracellular ROS resulting in a condition referred to as oxidative stress [26]. The eukaryotic cells are evolutionarily evolved to modulate the oxidant levels in a highly efficient manner by maintaining sufficient antioxidant levels, induction of new gene expression and protein modification, and tightly regulating their redox status within a narrow range [27]. However, in the event of persistent exposure to the oxidants and other toxic agents, excessive ROS are produced that are capable of causing oxidative damage to biomacromolecules such as peroxidation of membrane lipids, oxidation of amino acid side chains (especially cysteine), formation of protein-protein cross-links, oxidation of polypeptide backbones resulting in protein fragmentation, DNA damage, and DNA strand breaks [28, 29]. Out of all these events, the formation of lipid peroxidation products is highly damaging because it leads to widespread free radical reactions besides compromising the membrane integrity leading to loss of cell function and eventually results in severe cytotoxicity that may either lead to uncontrolled cell growth (neoplasia) or cell death (apoptosis) [30, 31].

**Figure 1:**
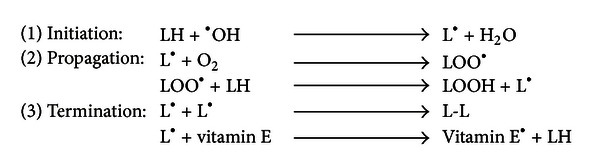
Three reaction steps in free radical initiated lipid (LH)—peroxidation leading to the formation of reactive lipid molecules (LOO^·^) that form lipid-derived aldehydes such as HNE, acrolein, MDA, and HHE.

The process of lipid peroxidation includes several chemical reactions or steps such as initiation, propagation, and termination (Figure 1) [32]. The first step of lipid peroxidation that is, initiation, includes hydrogen atom abstraction by free radicals such as hydroxyl (^·^OH), alkoxyl (RO^·^), peroxyl (ROO^·^), and HO_2_^·^. The initial reaction of ^·^OH with polyunsaturated fatty acids produces a lipid radical (L^·^), which in turn reacts with the molecular oxygen to form a lipid peroxyl radical (LOO^·^) in the propagation reaction. The LOO^·^ species then acquire a hydrogen atom from the neighboring fatty acid molecule and produce a lipid hydroperoxide (LOOH) and simultaneously generate a second lipid radical [32]. The LOOH can further be cleaved by reduced metals, such as Fe^++^, forming a lipid alkoxyl radical (LO^·^). In addition, LOOH may also break down into the reactive aldehyde products or LDAs including MDA, HNE, ONE, 4-HHE, and acrolein in the presence of reduced metals or ascorbate [13, 33, 34]. A chain reaction sets in which both alkoxyl and peroxyl radicals stimulate lipid peroxidation by abstracting additional hydrogen atoms from neighboring lipid molecules [33–35]. This results in the disruption of major chunk of cell membrane lipids that disturbs the assembly of cell membrane causing alterations in membrane fluidity and permeability, alterations of ion transport, and suppression of metabolic processes [36]. The final and third step is the termination which involves the formation of a hydroperoxide which is achieved by reaction of a peroxyl radical with *α*-tocopherol, a lipophilic chain-breaking molecule found in the cell membrane. Termination could also be achieved when a lipid radical (L^·^) reacts with lipid peroxide (LOO^·^) or when two peroxide molecules combine together and result in nonreactive species LOOL or hydroxylated derivative (LOH), respectively, which are relatively stable. Some of the lipid peroxides could also react with the membrane proteins that result in the termination [37, 38]. The propagation reaction is self-sustaining and continues unabated until the substrate is consumed or the termination reaction sets in by antioxidants or free radical quenchers [38]. Thus, being a self-sustaining process, lipid peroxidation amplifies the effects of the original free radical and results in extensive tissue damage.

**Figure 2:**
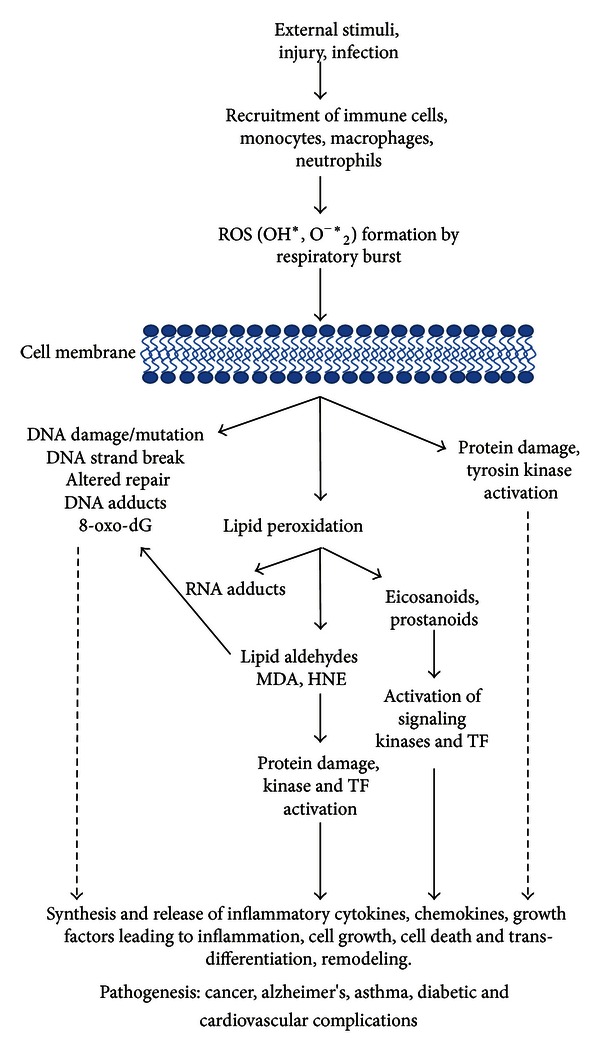
Contribution of lipid peroxidation-derived aldehydes in various disease complications.

Lipid peroxidation, an indicator of oxidative stress in cells and tissues, is a well-defined mechanism of cell injury. Peroxidation of membrane lipids has been shown to generate toxic and unstable biomolecules such as reactive carbonyl compounds including LDAs. These highly reactive electrophiles readily react with cellular proteins and nucleic acids, and also activate signaling cascade molecules and activate transcription factors thus causing inflammation (Figure 2). The cells have elaborate mechanisms to handle the excessive levels of LDAs which include enzymes that detoxify the LDA by reducing them to respective alcohols, for example, aldose reductase (AR) and aldehyde reductase or by oxidizing them to acids, for example, aldehyde dehydrogenase. Most of the unsaturated LDAs such as HNE and acrolein are also metabolized through forming GS-LDAs. Some of the glutathione-S-transferase (GST) isozymes such as human GSTA4-4 and GST5-8 have been shown to significantly catalyze the conjugation of HNE. Several studies indicate that regulation of enzymatic activity of GSTs could modulate the cytotoxicity associated with the HNE [39–41]. Further, LDAs also readily react with cellular glutathione (GSH) forming GS-LDA conjugates which further get reduced into GS-lipid alcohols and transported out of the cells. Recently, our laboratory has extensively presented evidence that AR-catalyzed GS-LDAs could mediate the activation of redox-sensitive transcription factors. 

## 3. NF-*κ*B and Cell Signaling 

NF-*κ*B is a family of transcription factors that regulate expression of numerous genes and play important roles in immune and stress responses, inflammation, and apoptosis [42–45]. There are five known proteins which come together as subunits to constitute the transcription factor NF-*κ*B. These are subunits p50 (derived from p105), p52 (derived from p100), p65 (RelA), c-Rel, and RelB. These subunits, ubiquitously expressed in mammalian cells and highly conserved across the species, can either form homodimer or heterodimer to form biologically active molecule of NF-*κ*B, which translocates to the nucleus upon phosphorylation and transcribes various genes [46]. Activation of NF-*κ*B via canonical or noncanonical pathways is an important mechanism to regulate the body's immune and inflammatory responses [47]. Various pathogens, oxidants, cytokines, chemokines, and growth factors either via specific receptors or general oxidative stress induce the molecular signals that eventually lead to activation of a redox-sensitive transcription factor NF-*κ*B [48]. Once activated either via canonical or noncanonical pathway, NF-*κ*B enters nucleus where it binds to the kB DNA binding sequence and transcribes various genes for cytokines, chemokines, and other inflammatory markers. The expression of a large number of genes involved in apoptosis, cell growth, survival, differentiation, and immune response is regulated by NF-*κ*B, which is associated with an array of diseases such as autoimmune, cancer, and inflammatory diseases.

## 4. Lipid Aldehydes in NF-*κ*B Mediated Cell Signaling

Increasing evidences suggest that aldehyde molecules generated endogenously during ROS-induced process of lipid peroxidation are involved in most of the pathophysiological effects associated with oxidative stress in cells and tissues [49, 50]. Though previously held view implied that lipid peroxidation products only elicit damage, more recently evolved paradigm suggests that their impact could be more varied and dependent upon factors including the species, concentration and the protein targets involved [51–53]. Increasing number of studies now implicate LDAs in regulating oxidant-induced cellular signaling [54–56]. The lipid aldehydes regulate cellular functions by interacting with the specific proteins covalently and non-covalently [57–59]. For example, protein adducts are formed through the reaction of lipid aldehydes with nucleophilic protein constituents, including amino acid residues such as cysteine, lysine, and histidine, resulting in Schiff base formation [60, 61]. Michael addition is another mechanism of protein adduction, where thiolate groups of cysteine residues react with electrophilic carbons present in *α*-*β* unsaturated carbonyls. The most simple oxidation products containing this reactive group, resulting from *β*-scission, include 4-HNE and acrolein. Please refer to few reviews available on the covalent reactivity of *α*-*β* unsaturated carbonyls with cellular biomolecules [57–61] for detailed information. 

LDAs have been shown to regulate various signaling pathways initiated by cytokines, chemokines, and growth factors. HNE has been shown to regulate many PKC isozymes depending upon its in situ concentration. For example, in rat hepatocytes low concentrations of HNE (0.1–1 uM) activate PKC beta1 and beta2 isozymes while higher concentrations of HNE (1–10 uM) inhibit PKC beta isozymes [62]. Further, PKC delta activity was inhibited by low concentrations of HNE (0.1 uM) and increased by high concentration of HNE (>1 uM; [63]). Although it is not clear how PKC isozymes are differentially regulated by HNE, it is possible that HNE could target upstream signals of PKC such as PLC and DAG which in turn may activate PKC isozymes. In addition to PKC, HNE can also regulate other kinases such as MAPK, ERK, and JNK indirectly by activating upstream kinases or directly by interacting with kinase active domains. Parola et al. [64] indicated that HNE could directly form conjugate with JNK which is responsible for histone modification and subsequent nuclear translocation. On the other hand, Song et al. [65] reported that HNE could activate JNK via activating an upstream kinase called stress-activated protein kinase kinase-1 (SPKK1). Similarly, HNE activates ERK via activating MEK1/2 and P38MAPK via activating MKK3/6 [66, 67]. However, it is not clear how HNE activates upstream kinases of ERK and MAPK. HNE could also activate receptor tyrosine kinases such as EGFR and PDGFR through direct conjugation [68]. 

**Figure 3:**
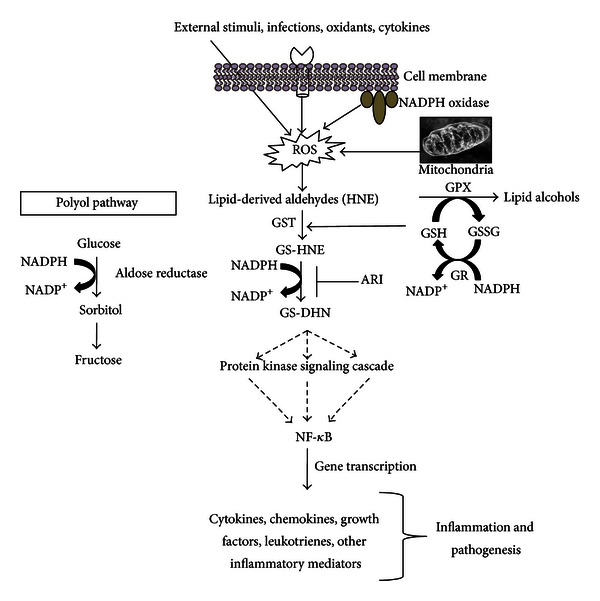
Regulation of lipid aldehyde-induced inflammatory signaling by aldose reductase.

The cell signaling pathways activated by LDAs include noncovalent mechanisms that involve binding to a protein receptor and covalent mechanisms that modify protein kinases directly. The direct interaction of LDAs with protein kinases such as SRC and PLC could change cellular calcium signaling and alter in-situ cation mobilization leading to activation of the caspases involved in cell death. Vatsyayan et al. [6] have shown that HNE besides being cytotoxic, at lower doses it triggers phosphorylation of epidermal growth factor receptor (EGFR) and activation of its downstream signaling components ERK1/2 and AKT which are known to be involved in cell proliferation. Similarly, Chaudhary et al. [69] concluded that HNE could evoke signaling for defense mechanisms to self-regulate its toxicity and simultaneously may affect multiple signaling pathways through its interactions with membrane receptors and transcription factors/repressors [57]. Dwivedi et al. [70] suggested that HNE has concentration-dependent opposing effects to cell death and growth. This report indicates that constitutive levels of HNE are needed for normal cell functions and low levels of HNE promote proliferative machinery while high levels promote apoptotic signaling. Further, they suggested that HNE can modulate ligand-independent signaling by membrane receptors such as EGFR or Fas (CD95) and may act as a sensor of external stimuli for eliciting stress-response, suggesting a key role of HNE in cellular signaling [70]. Furthermore, our laboratory has presented numerous evidences [71–73] that GS-LDAs when enzymatically reduced by AR, become important secondary messengers to activate signaling molecules that eventually leads to activation of NF-*κ*B via yet unknown mechanism (Figure 3). The regulation of NF-*κ*B activation is the key for LDAs to modulate various inflammatory pathologies. Since NF-*κ*B transcribes various proinflammatory cytokines, growth factors, cell survival proteins, structural proteins, and apoptotic proteins, regulation of intracellular concentrations of LDAs could be novel strategy to prevent inflammatory complications. Indeed, recent studies indicate that enzymes that regulate LDAs generation or participate in their metabolism could actually prevent pathological effects of LDA-induced inflammatory complications [2–5, 73]. 

## 5. Activation of NF-*κ*B under Oxidative Stress

Increased oxidative stress is a hallmark of inflammatory diseases such as those caused by infections, xenobiotics, environmental pollutants, and those of autoimmune etiology, and ROS is an essential mediator of intracellular signaling under a variety of conditions [74–79]. Several lines of evidence suggest that ROS mediates the activation of redox-sensitive transcription factors such as NF-*κ*B and AP1, which in turn stimulate the expression of an array of inflammatory cytokines and chemokines genes [80, 81]. These evidences largely suggest the use of a variety of antioxidants that inhibit NF-*κ*B activation and also antioxidant enzymes which are overexpressed as a counter mechanism to bring down the NF-*κ*B activation. Excessive and unrestrained production of inflammatory mediators causes cytotoxicity in an autocrine and paracrine manner. Among various redox-sensitive transcription factors such as NF-*κ*B, AP1, CREB, ERF2, NFAT, and ATF2 that are activated by increased ROS levels, NF-*κ*B has been extensively studied and is implicated in many oxidative stress-induced inflammatory diseases [45–49]. Activation of NF-*κ*B under oxidative stress has been noticed in a number of inflammatory complications. However, how exactly ROS activates various protein kinases upstream to NF-*κ*B is not known clearly. In the preceding section, we presented evidence that suggests that ROS-generated HNE and other LDAs may directly or indirectly activate upstream kinases including tyrosine receptor kinases [6, 68].

We and others have also presented many evidences which implicate ROS-induced lipid peroxidation products such as lipid aldehydes in the activation of signaling cascade that eventually activates NF-*κ*B [70, 73, 82, 83]. Indeed, ROS-induced lipid peroxidation has been proposed to be major contributor in the pathophysiology of many inflammatory disorders. HNE, generated by a number of oxidative stress conditions inducing etiologies including bacterial infection, xenobiotics, environmental pollutants, and autoimmune disorders, is one of the highly abundant and toxic lipid aldehydes [84]. Acrolein is another highly reactive species generated both exo- and endogenously. Depending upon their respective concentrations both HNE and acrolein elicit phenotypically varied outcomes. Both are known to activate various upstream kinases including MAPK and PKC [71, 82, 85–89], thus enzymes that either regulate or metabolize HNE could be mediators of oxidative stress signals. Modification of multiple cytoskeletal proteins and the activation of MAPKs and ROS-sensitive transcription factors by HNE metabolizing enzymes have been demonstrated by various investigators [71, 82]. Further, growth factors and cytokine-induced increased generation of ROS could be an essential step for cell growth because overexpression of antioxidants such as catalase and super oxide dismutase (SOD) or treatment with antioxidants such as N-acetylcysteine (NAC) are known to diminish growth factor and cytokine-stimulated cell growth or death [90, 91]. Many studies have indeed indicated that ROS themselves can act as toxic messengers that activate NF-*κ*B and affect the cellular functions of growth factors, cytokines and other molecules [92, 93]. These evidences clearly indicate that various stimulants and oxidants activate redox-sensitive transcription factors including NF-*κ*B by generating ROS which in turn generate other secondary messengers such as lipid aldehydes that phosphorylate upstream signaling kinases. 

## 6. Modulation of Oxidative Stress and Lipid Aldehydes Signals by Antioxidants

In oxidative stress-induced inflammatory diseases, antioxidant status of the tissues undergoes severe alteration leaving the cells overexposed to oxidative free radicals. The lipid peroxidation caused by increased ROS continues unabated leading to generation of further more free radicals and in this way a vicious cycle continues which results in the establishment and progression of the disease [94]. The cells are endowed with antioxidant system which includes (a) SOD that catalyzes the breakdown of the superoxide anion into oxygen and hydrogen peroxide; (b) catalase, which catalyzes the conversion of hydrogen peroxide to water and oxygen, and (c) peroxiredoxin, which catalyzes the reduction of hydrogen peroxide, organic hydroperoxides, and peroxynitrate to tackle oxidative bouts [95, 96]. In addition, the cells also contain glutathione system which includes glutathione, glutathione reductase, glutathione peroxidases, and glutathione *S*-transferases, which maintains redox balance and protect from oxidative challenges [97, 98]. However, in the overwhelming oxidative stress these mechanisms become ineffective and cellular homeostasis disrupts [98]. Many investigators have demonstrated that supplementation of antioxidants in the experimental animal models of oxidative stress-generated inflammatory pathologies successfully blocks the inflammatory changes [98–100]. Many antioxidants that inhibit oxidative stress signaling pathways are known to be effective in halting the inflammation in experimental animal models. For example, Vitamin C and E, N-acetylcysteine (NAC), Lipoic acid, GSH, carotenoids and flavonoids from plants, and melatonins, have been shown to be effective in experimental models of many diseases including cardiovascular, neurodegenerative, infection, diabetic complications, autoimmune, and allergic complications. A number of antioxidants have undergone clinical trials [101–107] to prevent disease pathologies including cardiovascular and cancer. These antioxidants however have not been successfully translated for the clinical use because at the clinical doses they become prooxidants and result in serious side effects. This restricts the use of antioxidants as therapeutic drugs and leaves them as prophylactic or preventive drugs only. In this scenario, a new drug which can be both antioxidative as well as anti-inflammatory that could regulate both ROS, and LDAs-induced signals could be a better approach to prevent inflammatory complications. Our results from the use of AR inhibitors in different preclinical models indicate that AR inhibitors such as fidarestat could be such a drug that can be used in the amelioration of inflammatory diseases including cardiovascular, sepsis, infection and autoimmune-induced uveitis, allergic asthma, cancer and metastasis, and angiogenesis [73, 108]. These encouraging observations have helped us to develop a highly specific and potent AR inhibitor, fidarestat, which has been tested in the phase-iii clinical studies for diabetic neuropathy and found to be safe for human use, as a potential antioxidative, anti-inflammatory, antiangiogenic, antimitogenic, and chemopreventive drug for preventing inflammatory diseases such as allergic asthma, colon cancer, and uveitis.

## 7. Role of AR in Mediation of Lipid Aldehydes Signals

It is well known that AR is overexpressed under oxidative stress initiated by various cytokines, growth factors, bacterial endotoxins such as lipopolysaccharides (LPS), and high glucose. Overexpression of AR increases the turnover of ROS-generated LDAs [73, 108]. The LDAs readily react with cellular GSH and form GSH-LDA conjugates which are excellent AR substrates [109, 110]. Since ROS is known to mediate and regulate intracellular signaling under diverse conditions, some of the effects of ROS can be mediated by ROS-derived LDAs and their GSH conjugates. We have systematically investigated the involvement of lipid aldehydes and their GSH conjugates in the mediation of signaling cascades that play important role in the pathophysiology of a number of diseases. The results from our studies indicate that reduction of LDAs and their GSH conjugates by AR is essential for transduction of cytotoxic signals [82–84]. This was firmly confirmed by various evidences including that AR has poor affinity for glucose (Km of 50–100 mM), and in normal conditions only a small percentage (3%) of glucose is metabolized by AR [111]. Further, the kinetic and structural properties of AR are unlike those of other glucose-metabolizing enzymes [112, 113]. The low Km for catalysis of carbohydrate reduction is probably due to high hydrophobicity of the substrate binding domain of AR which essentially prevents efficient reduction of glucose by AR and suggests that hydrophobic aldehydes could likely be the preferred substrates. Indeed, we have unequivocally demonstrated that AR efficiently catalyzes lipid aldehydes and their GSH conjugates [109, 110]. For example, recombinant human AR has been shown to catalyze the reduction of a large series of saturated and unsaturated aldehydes with 1000-fold higher efficiency when compared to glucose [109–112]. Medium- to long-chain (C-6 to C-18) aldehydes, which are generated during lipid peroxidation, are most efficiently catalyzed by AR [109]. In particular, the catalytic site of AR has more affinity towards GS-aldehyde conjugates than parent aldehydes. This is confirmed by site-directed mutagenesis studies showing that AR active site has amino acid residues which efficiently bind with glutathione moiety [109]. Further, molecular modeling of AR-GSH analog crystal structure confirmed that AR has a specific GS-aldehyde binding site at its catalytic site [112]. Thus, these observations firmly support that physiological role of AR could be the reduction of LDAs besides glucose metabolism in polyol pathway. 

Since LDAs are known to modulate the cellular function by regulating the oxidative stress signals mediated by NF-*κ*B and AP1 [114–118], we postulated that AR regulates cellular functions by modulating oxidative stress signals. Our claim is supported by the studies demonstrating that AR inhibition prevents HNE-, growth factor-, and cytokine-induced cytotoxicity in a variety of cultured cells [73, 108]. Further, our studies also demonstrated that inhibition of AR prevents endotoxin-, allergen-, cytokine-, and growth factor-induced activation of NF-*κ*B signals (Figure 3). In all these conditions, increased ROS levels, lipid peroxidation, and subsequent formation of lipid aldehydes play a major role in disrupting the cellular homeostasis. Thus, our observations present a basis for the novel role of AR in the pathophysiology of various inflammatory disease processes mediated by LDAs.

Besides the novel role implicating AR in mediating signaling of the ROS-derived lipid aldehydes in inflammatory pathologies, the exact role of AR in the mediation of cellular signaling is not yet clear. How AR-catalyzed product of GS-LDAs activates PKC and PLC isozymes still needs to be investigated. Nevertheless, we have shown that AR-catalyzed reduced product of GS-DHN activates various kinases including PLC, PKC, and PI3K in cultured cells. Activation of these kinases eventually results in the activation of redox-sensitive transcription factors which transcribe various inflammatory genes responsible for disease progression. The involvement of AR-catalyzed reaction products in eliciting oxidative stress-induced cytotoxicity and inflammation is more obvious given the fact that inhibition of AR prevents the increased synthesis of inflammatory cytokines and chemokines by various oxidant stimuli such as LPS, cytokines, growth factors, and high glucose. This is further substantiated by our demonstration that HNE, GS-HNE, and AR-catalyzed reduced product of GS-LDAs, that is, GS-DHN promote, VSMC growth in vitro [71]. AR inhibition by pharmacological inhibitors or ablation by siRNA prevents HNE- and GS-HNE-induced VSMC proliferation but has no effect on the GS-DHN-induced changes. These studies thus suggest that the reduced forms of lipid-aldehyde glutathione conjugates (such as GS-DHN) could be involved in the oxidative stress-induced inflammatory signaling. Further studies are required to investigate the exact mechanism of GS-DHN-induced activation of upstream kinases that results in transcription of inflammatory genes. Nevertheless, our observations have opened up a new area of research in understanding the role of AR in the mediation of oxidative stress signaling in a number of inflammatory diseases.

## 8. Current and Future Developments

Oxidative stress-induced generation of lipid aldehydes has been observed in many inflammatory complications including cardiovascular disorders, bacterial sepsis, cancer, and asthma, which present enormous clinical challenges worldwide. Many researchers have presented the immense importance of this aspect of pathophysiology, and therefore there is an urgent need for development of new therapeutic strategies targeting the intervention in lipid aldehyde-mediated inflammatory signals in these diseases. However, a more precise elucidation of lipid aldehyde-induced inflammatory signaling is crucial for understanding the pathophysiology of multiple diseases including infections, atherosclerosis, autoimmune, and cancer. Based on these elucidations, a better therapeutic intervention could be developed to contain the inflammatory responses in patients. Our extensive research during recent years has identified that oxidative stress-induced LDAs and their GS-conjugates catalyzed by AR play a major role in the mediation of NF-*κ*B-induced inflammatory signals via PLC/PKC/IKK/MAPK pathways. We have demonstrated that in experimental animal models, inhibition of GS-LDA metabolizing enzymes, specifically AR, prevents inflammatory diseases such as uveitis, sepsis, colon cancer, atherosclerosis, and allergic asthma. These results have delineated a novel mechanism regulating inflammation and have laid foundation for future studies that could result in clinical application of AR inhibitors. Further, a better understanding of the role of AR-catalyzed LDAs and their GSH-conjugates in the signalosome and inflammasome signaling pathways will better reveal underlying pathophysiological events in various inflammatory diseases.

## Abbreviations

AR: Aldose reductase
ARI: AR inhibitor
ROS: Reactive oxygen species
LDAs: Lipid peroxidation-derived aldehydes
MDA: malondialdehyde
HNE: 4-hydroxynonenal
GSH: Reduced glutathione
GS-LDAs: Glutathione-lipid-derived aldehydes
GS-HNE: Glutathione-HNE conjugate
GS-DHN: Glutathione-1,4-dihydroxynonene conjugate
LPS: Lipopolysacharides
NADPH: Reduced nicotinamide adenine dinucleotide phosphate
PKC: Protein kinase C
PLC: Phospholipase C
MAPK: Mitogen-activated protein kinase
NF-*κ*B: Nuclear factor kappa B
AP-1: Activator protein 1
CREB: cAMP response element-binding protein
VSMC: Vascular smooth muscle cells
SOD: Superoxide dismutase
NAC: N-acetylcysteine
AMD: Age-related macular degeneration
EGFR: Epidermal growth factor receptor.
